# Use of Fluorescence Imaging in Liver Transplant Surgery

**DOI:** 10.3390/jcm13092610

**Published:** 2024-04-29

**Authors:** Alvaro Ducas, Alessandro Martinino, Lorna Astrid Evans, Emiliano G. Manueli Laos, Francesco Giovinazzo, on behalf of the SMAGEICS Group

**Affiliations:** 1 Department of Surgery, University of Illinois at Chicago, Chicago, IL 60607, USA; alvaroducas@gmail.com (A.D.);; 2 Department of Surgery, Duke University, Durham, NC 27710, USA; 3 Department of Surgery, Mayo Clinic, Jacksonville, FL 32224, USA; evans.lorna@mayo.edu; 4 Department of Surgery, Fondazione Policlinico Universitario Agostino Gemelli IRCCS, 00168 Rome, Italy; 5 Department of Surgery, Saint Camillus Hospital, 31100 Treviso, Italy; 6 Department of Surgery, UniCamillus-Saint Camillus International University of Health Sciences, 00131 Rome, Italy

**Keywords:** liver transplant, ICG, fluorescence, artificial intelligence

## Abstract

Liver transplant surgery is a complex procedure that demands high knowledge of surgical anatomy and the precise recognition and preservation of structures. To address this, the use of fluorescence imaging has facilitated the identification of anatomical structures such as biliary ducts, arteries, and liver segmentation. Indocyanine green is among the most commonly utilized fluorescent agents, not just during surgery but also in the pre- and postoperative phases, where it is used to assess graft failure by measuring the plasma disappearance rate. New advancements such as artificial intelligence paired with fluorescence imaging have the potential to enhance patient outcomes. Additionally, technologies such as augmented reality and mixed reality could be integrated into surgical procedures, broadening the scope of possibilities for improving patient safety.

## 1. Introduction

Successful outcomes in liver transplantation correlate significantly with accurate identification and meticulous preservation of crucial anatomical structures, as evidenced by improved graft function and reduced postoperative complications. One of the tools available to surgeons is fluorescence imaging, which, during liver transplantation, demonstrably enhances surgical techniques by facilitating real-time visualization of key anatomical structures and improves postoperative care by enabling quantitative assessment of graft function. This last function potentially leads to earlier detection and intervention in cases of graft failure [1,2]. Fluorescence imaging technology has revolutionized the way surgeons approach liver transplants, improving patient safety and surgical outcomes. In this article, we will explore the use of fluorescence imaging in liver transplant surgery, its benefits, and its impact on this field.

Clinical fluorescence imaging has been used in surgery for many years. It makes use of contrast agents that are fluorophores, which absorb light in the near-infrared region of the spectrum (790–805 mm) and then emit fluorescence at 835 mm. The light then is detected by a sensor and projected on a monitor in real time [3]. The most common agent used for clinical fluorescence imaging is indocyanine green (ICG), which has been used since 1960 and approved by the Food and Drug Administration (FDA) for clinical and research use in humans since 1956 [4]. Indocyanine green is an amphiphilic, tricarbocyanine dye reconstituted in aqueous solution of pH 6.5 for intravenous injection in patients. When ICG reaches the intravascular space, it binds to plasma proteins, specifically albumin and alpha and beta lipoproteins. Of the injected ICG, 98% is bound to plasma proteins and remains in the intravascular space for a longer time; the remaining 2% is free in the serum until hepatic uptake and bile excretion [5,6]. This compound has been widely applied to surgical procedures such as lymph node dissection, arterial bypass grafting, cholecystectomy (cystic duct recognition), pancreatoduodenectomy, colorectal resection, and bowel anastomotic perfusion assessment, especially in bariatric surgery and nephrectomy [7]. Furthermore, the use of ICG in reconstructive surgery helps in the assessment of perfusions of locoregional and free flaps [8]. Although it is generally well tolerated, uncommon adverse effects associated with IGC administration include nausea and cephalalgia (0.15%), with rare occurrences of life-threatening anaphylactic reactions (0.05%). Consequently, due to potential cross-reactivity, ICG is contraindicated in patients with a history of iodine hypersensitivity. However, it exhibits a favorable safety profile in individuals with end-stage liver or kidney disease [3].

**Figure 1:**
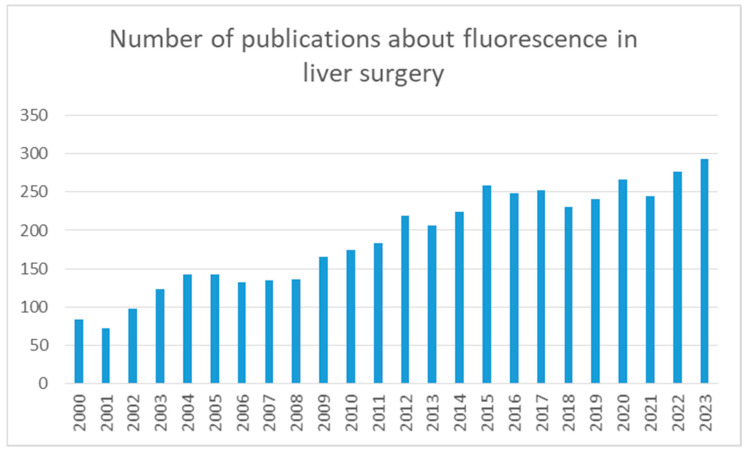
Search results in PubMed regarding the use of ICG in liver surgery.

This upward trend in ICG utilization within hepatobiliary surgery is demonstrably evident over the past two decades, as documented in Figure 1. Significantly, the annual publication output has more than tripled compared to what it was 20 years ago.

## 2. ICG in Hepatobiliary Surgery

The use of ICG in hepatobiliary surgery has been well described over the years. Table 1 shows a selection of publications that studied the benefits of using fluorescence imaging in this type of procedure.

The annual incidence of laparoscopic cholecystectomy globally reaches 1.2 million, highlighting the widespread need for effective surgical strategies in biliary disease management [9]. This, coupled with the growing burden of liver tumors, underscores the increasing significance of intraoperative imaging modalities like indocyanine green (ICG) fluorescence, which empowers surgeons with real-time visualization and data-driven decision-making capabilities during complex procedures [2,10]. Its usefulness is explained by the fact that ICG exhibits a high affinity for albumin in plasma, facilitating its delivery to the liver. Following hepatic uptake by hepatocytes, ICG undergoes active biliary excretion, rapidly reaching peak concentration within 2 h and demonstrating a sustained insignificant presence for the subsequent hour [11].

One of the most benefited procedures is laparoscopic cholecystectomy; though it is minimally invasive, difficulties are often presented in visualizing the intricate extrahepatic biliary anatomy, increasing the risk of misidentification and complications [12]. In 2008, Ishizawa introduced a turning point with the introduction of fluorescence cholangiography (FC) in this procedure. By employing FC, surgeons gained a novel tool to enhance real-time visualization of the crucial biliary structures, paving the way for safer and more precise laparoscopic cholecystectomies [13]. ICG fluorescence imaging represents a transformative advancement in laparoscopic cholecystectomy, replacing the potentially hazardous practice of intraoperative cholangiography with a safer and more efficient alternative. ICG non-invasively delineates the critical Calot’s triangle structures, obviating the need for cystic duct puncture and eliminating radiation exposure, thereby contributing to superior surgical outcomes [14].

In addition, ICG fluorescence imaging exhibits promising capabilities for detecting subcapsular liver tumors within 8 mm of the surface, offering a valuable tool during major hepatectomies and donor hepatectomies [15]. The use of ICG illuminates the biliary tract by leveraging the inherent biliary excretion of this agent following intravenous administration. Consequently, clear visualization of the biliary anatomy becomes evident approximately 30 min post injection, facilitating the precise identification of bile duct division points, thereby enhancing surgical planning and potentially minimizing complications [16]. This intraoperative identification of hepatic tumors relies on the differential uptake and retention of ICG by neoplastic tissue. Compared to normal hepatocytes, which rapidly excrete ICG, hepatocellular carcinomas (HCCs) and other well-differentiated malignancies demonstrate enhanced ICG fluorescence due to impaired bile salt export pump function [17,18]. This differential retention of ICG allows real-time tumor visualization during hepatectomy, facilitating margin delineation and potentially minimizing the risk of incomplete resection. ICG fluorescence allows a high sensitivity of 90% or higher when localizing a tumor. Additionally, this is achieved by the administration of 0.25–0.5 mg/kg of ICG from 12 to 14 h prior to the surgical procedure [19,20,21].

Another use in hepatobiliary surgery is in intraoperative hepatic segmentation, facilitating the precise delineation of intersegmental planes. This technique extends beyond visualizing surface anatomy, providing surgeons with real-time guidance during parenchymal dissection throughout hepatectomy procedures, even on raw liver surfaces [17]. Consequently, ICG fluorescence imaging enhances the spatial orientation and decision-making process during liver resection, potentially leading to improved surgical outcomes. Ishizawa et al. pioneered the utilization of ICG for positive and negative staining during liver resection. In positive staining, primarily employed during open surgery, ICG is directly injected into a segmental portal vein branch visualized through intraoperative ultrasonography. This results in fluorescence emission from the perfused liver segment due to ICG uptake by hepatocytes. Conversely, negative staining involves temporary occlusion of the portal pedicle supplying the target segment. Subsequent ICG administration then leads to fluorescence solely in non-occluded segments, highlighting the target for resection [22]. This previously described technique is used during liver resections and during living liver donor transplantation.

## 3. Liver Transplantation

Successful liver transplantation relies on the accurate evaluation of graft function and perfusion parameters. In this context, ICG emerges as a valuable tool, offering real-time data on graft hepatocyte function and blood flow dynamics. Consequently, ICG empowers surgeons with critical intraoperative insights, facilitating informed decision-making throughout the transplant process. Following the description of ICG applications in liver surgery, a demonstrably significant upward trend has been observed in the utilization of fluorescence imaging within the field, as evidenced by the increasing volume of research publications. Notably, within liver transplantation, ICG has emerged as a versatile tool across various stages of the procedure: preoperatively in assessing donor and graft viability; intraoperatively in evaluating vascular patency, liver perfusion, delineating the demarcation line during donor hepatectomy, and biliary anatomy; and postoperatively in monitoring graft function and detecting early graft failure and complications [3].

Pure laparoscopic donor hepatectomy (PLDH) was performed for the first time at Seoul National University Hospital (SNUH) in 2015. Kim et al. described at the same hospital the use of ICG for exact midplane identification during laparoscopic donor hepatectomy. The technique consists of clamping the right/left hepatic artery and portal vein, intravenously injecting ICG, and with a near-infrared camera demarcating the exact plane for dissection [23,24].

One of the primary uses of ICG in liver transplant surgery is to assess if the donor liver is suitable before transplantation. By injecting ICG into the donor’s bloodstream, surgeons can evaluate the liver’s function and blood flow using ICG clearance parameters, helping them to determine whether the organ is suitable for transplantation. This preoperative assessment can help reduce the risk of complications and improve the overall success of the procedure [25]. Then, during the intraoperative phase, ICG can be used to assess the graft’s function and blood flow. This real-time information allows surgeons to monitor the liver’s condition and make necessary adjustments to ensure its proper function. Additionally, ICG can help identify any potential complications, such as poor blood flow or inadequate liver function, allowing for timely intervention and improved patient outcomes. After the transplantation, ICG can be used to continue monitoring the graft’s function and blood flow during the postoperative period using ICG kinetics during the following days after transplantation; this includes the evaluation of ICG clearance just like in the preoperative phase [25]. This can help detect any issues early on and guide the management of the patient’s care [1].

The methods using ICG can be divided into those measuring the clearance from plasma (which detects graft’s failure in an early phase) and those detecting its presence using near-infrared cameras during the surgical procedure (anatomical recognition). Among the factors that have shown to affect graft utilization and function we can consider advanced donor age, hypernatremia, prolonged warm ischemia time, vasopressor requirement, and donation after cardiac death [26].

### 3.1. ICG Clearance

Measuring the levels of ICG in plasma over time (ICG clearance) can be used as a marker of liver function providing useful objective information. Indocyanine green is administered intravenously and it is then taken by the hepatocytes and excreted in the bile ducts. The disappearance rate is measured in a blood sample or using a pulsidensiometric method (plasma disappearance rate, PDR). The cut-off PDR value of >14%/min allows safe major liver resections [27,28]. An increased incidence of graft failure has been reported with PDR < 11%/min [29]. Following intravenous administration, plasma ICG concentration is traditionally measured using blood spectrophotometry, considered the gold-standard method. Pulse transcutaneous ICG measurement has emerged as a potential alternative, demonstrating promising results with correlation coefficients ranging from 0.8 to 0.95. However, further investigation and validation are necessary before it can supplant the well-established accuracy and reliability of blood spectrophotometry [30].

The use of ICG clearance in donor assessment gives objective information before procurement, assisting in decision-making around the acceptance of marginal grafts. The dose used in this case is 0.5 mg/kg. During the procurement phase, ICG clearance is useful for the assessment of the liver quality during ex vivo machine perfusion or during laparoscopy in a dose of 10 mg. [3] Furthermore, it can be used as well for post-transplant assessment of graft function. This is supported by many studies that show a correlation between the poor ICG clearance with early allograft dysfunction (EAD). Jalan et al. observed a lower ICG clearance at 18–23 h post transplant (<200 mL/min) in patients that died, required retransplantation, or had a complicated postoperative course [31]. The indocyanine green dye plasma disappearance rate (ICG-PDR) is a simple validated tool for liver function assessment. Cherchi et al. determined the ICG-PDR before graft retrieval and 24 h after transplant, evaluating postoperative early allograft dysfunction using the MEAF (Early Allograft Function) score. MEAF grades the severity of liver graft dysfunction based on bilirubin, international normalized ratio, and alanine aminotransferase within three days post transplant. It is a prognostic tool for 3-, 6- and 12-month patients and graft survival [32].

Cherchi et al. also conducted a retrospective single-center study comparing the indocyanine green dye plasma disappearance rate (ICG-PDR) before graft retrieval and 24 h after transplant in orthotopic liver transplantation. These data were compared with the MEAF model of graft dysfunction. After 36 ICG-PDR measurements they found a direct association between the variation rate of the donor–recipient ICG-PDR and MEAF, making this an easy and repeatable bedside measurement for the estimation of perioperative liver graft dysfunction [32].

### 3.2. ICG Fluorescence

The use of ICG fluorescence imaging facilitates intraoperative decision-making, enhancing patient safety. For this purpose, clinical fluorescence imaging utilizes a contrast agent that absorbs and emits light at longer wavelengths. The emitted light is detected by a sensor and projected on a monitor in real time. The most-used agent for clinical fluorescence imaging is ICG. It has a peak spectral absorption of 800–810 nm in plasma or blood and an emission peak of 835 nm. The half-life of ICG in blood is 2.5–3 min, and the maximum recommended dose in adults is 2 mg/kg body weight. The rate of allergic reaction has been reported at 0.05% [33,34].

ICG fluorescence is used to assess liver transplant inflow vessels in real time to confirm blood flow in the hepatic artery and portal vein [35]. ICG fluorescence has been used as well to assess areas of veno-occlusion within the liver after living-donor LT where either the donor or recipient is deprived of the middle hepatic vein outflow. The use of intraoperative indocyanine green (ICG) fluorescence imaging also facilitates the real-time assessment of liver perfusion, enabling surgeons to identify and manage portal vein thrombosis with enhanced accuracy and efficiency [36].

The real-time visualization of the biliary tree by a near-infrared fluorescent imaging camera is another important use. This allows the identification, during a living donor hepatectomy for liver transplantation, of the appropriate bile duct division which is optimal for both donor and recipient [37]. A transcystic approach is preferred at open surgery (ICG 0.025 mg/mL) while a systemic intravenous technique is preferred during laparoscopic techniques (ICG 0.05–0.1 mg/kg).

Mizuno et al. described the use of fluorescence cholangiography in a live-donor liver transplantation (LDLT) who underwent open left hepatectomy. After the hilar plate was isolated, intrabiliary ICG injection allowed visualization of the left hepatic duct and posterior branch of the right hepatic duct, guiding a cutting line of the bile duct that fits both the donor and the recipient [34]. Hong et al. also demonstrated that fluorescence imaging with ICG helps in the delineation of the biliary tree around the hilar plate, allowing real-time identification of the optimal bile duct division points during laparoscopic LDLT [37].

There are also potential limitations associated with the use of ICG in liver transplant surgery. For example, the dye’s fluorescence can be affected by factors such as obesity and jaundice, which may limit its accuracy in some patients. Imagi et al. described obesity as an important factor that can prevent the identification of biliary structures under near-infrared imaging [38]. Ankersmit et al., however, found no difference regarding biliary structure visualization in obese patients [39]. It is clear that thicker tissue covering the structures to identify will interfere with fluorescence imaging requiring a higher penetration rate but there are other factors that influence the success rate like inflamed tissue.

## 4. Future Perspectives

By enabling real-time, intraoperative visualization of anatomical structures, fluorescence imaging has significantly improved surgical outcomes by facilitating more precise resections, minimizing complications, and optimizing patient recovery [40]. Additionally, precise tumor localization enhances complete resection and reduces residual disease, while detailed anatomical identification guides complex procedures and improves decision-making. Furthermore, fluorescence imaging offers comprehensive perfusion assessment for evaluating tissue viability and guiding vascular reconstructions and facilitates accurate lymphatic mapping for sentinel lymph node identification and cancer staging [41].

In addition, there are more technologies to develop and study; indeed, the integration of artificial intelligence (AI) in conjunction with fluorescence imaging holds great promise. By leveraging machine learning algorithms, AI can analyze images taken from previous surgeries and CT scans performed on a patient before the surgery to recognize and label anatomical structures in real time. This integration can further enhance surgical precision and potentially reduce the risk of complications during liver transplant surgery. Moreover, the use of AI in conjunction with fluorescence imaging could also provide valuable insights and predictive analytics for surgeons, enabling them to make more informed decisions during surgery and potentially improving patient outcomes.

Furthermore, augmented reality (AR) has emerged as a transformative technology within image-guided surgery, significantly enhancing its utility. Leveraging artificial intelligence (AI), AR superimposes virtual models onto the patient’s real-time intraoperative view, facilitating the recognition and localization of crucial anatomical structures [42]. This synergistic integration of preoperative patient data with real-time surgical images creates a mixed-reality environment, providing surgeons with invaluable real-time guidance and potentially optimizing surgical precision and efficiency.

As technology develops, there is ample reason for more research and development into the possible application of AI-powered fluorescence imaging in liver transplant surgery. The future integration of these technologies appears to hold significant potential for enhancing the safety and efficacy of liver transplant procedures.

## 5. Conclusions

Liver transplantation surgery is a complex procedure, requiring high knowledge in surgical anatomy, structure preservation, and surgical skills. The application of fluorescence imaging in liver transplant surgery has been shown to be a useful technique for enhancing the visibility of structures and achieving better surgical outcomes. Its accuracy in locating arteries, bile ducts, and other crucial structures may reduce complications and improve patient outcomes. ICG is the best option available to help assess the patient during the peri-, intra-, and postoperative phases. Through intraoperative blood flow and tissue perfusion assessment, ICG helps surgeons make better decisions in real time and enhances patient outcomes while providing a clearer picture of biliary anatomy and liver tumors. Uses of ICG include measuring the clearance from plasma and detecting the presence of ICG using near-infrared cameras during the surgical procedure; early liver graft failure can be detected as well, which saves time while improving patient outcomes. Variables, including thicker tissue, such as that found in patients with obesity, or inflammatory tissue, may limit the dye’s accuracy and affect its fluorescence.

New technologies such as artificial intelligence can work in conjunction with fluorescence imaging, providing valuable tools for insights and predictive analytics for surgeons to improve patient outcomes. Image-guided surgery incorporating AI, augmented reality, and fluorescence imaging will collectively overcome the limitations inherent to each technique when used individually. Further studies are still required, but these technologies are among the most promising in terms of facilitating surgical decision-making during the perioperative, intraoperative, and postoperative periods.

## Figures and Tables

**Table 1:** Selection of publications regarding the use of ICG in hepatobiliary surgery.

Author	Year	Patients	Dose	Time after Injection	Procedure
Ishizawa	2009	49	0.5 mg/kg	3 days	Liver tumor identification
Ishizawa	2009	1	2.5 mg	2 h	Intraoperative biliary anatomy visualization
Morita	2013	58	0.5 mg/kg	3 to 28 days	Preoperative hepatic function
Kudo	2014	17	0.5 mg/kg	2 weeks	Visualization of subcapsular hepatic malignancy
Osayi	2015	82	6 mg	60 min	Intraoperative biliary anatomy visualization
Kawaguchi	2015	1	0.93 mg	Intraoperative	Intraoperative hepatic perfusion evaluation in liver graft
Panaro	2017	6	0.5 mg/kg	47 s	Intraoperative fluorescence angiography
Terasawa	2017	41	0.5 mg/kg	3 days	Liver tumor identification
Tang	2017	90	0.5 mg/kg	6 h	Donor graft quality prediction
Kim	2021	46	0.025 mg/kg	85.6 min	Midplane demarcation for laparoscopic hepatectomy
Symeonidis	2024	80	0.3 mg/kg	6 h	Intraoperative biliary anatomy visualization
